# Effects of pre-operative oral carbohydrates on insulin resistance and postoperative recovery in diabetic patients undergoing coronary artery bypass grafting: study protocol for a prospective, single-blind, randomized controlled trial

**DOI:** 10.1186/s13063-022-07042-w

**Published:** 2022-12-30

**Authors:** Shicheng Zhang, Lixian He, Yiping Yu, Xin Yuan, Tao Yang, Fuxia Yan, Fei Xu, Yan Zhang, Shiwei Pan, Huaijun Zhang, Zujun Chen, Lu Xie, Rong Wu, Wei Feng, Yuntai Yao, Evidence In Cardiovascular Anesthesia (EICA) Group

**Affiliations:** 1 Department of Adult Cardiac Surgery, Fuwai Hospital, Chinese Academy of Medical Sciences & Peking Union Medical College/National Center for Cardiovasular Diseases, Beijing, China; 2 Department of Anesthesiology, Fuwai Yunnan Cardiovascular Hospital, Kunming, Yunnan Province China; 3 Department of Anesthesiology, Fuwai Hospital, Chinese Academy of Medical Sciences & Peking Union Medical College/National Center for Cardiovascular Diseases, Beijing, China; 4 Department of Intensive Care Unit, Fuwai Hospital, Chinese Academy of Medical Sciences & Peking Union Medical College/National Center for Cardiovascular Diseases, Beijing, China

**Keywords:** Preoperative carbohydrates, Diabetes mellitus, Off-pump coronary artery bypass grafting, Insulin resistance

## Abstract

**Background:**

Preoperative carbohydrates (CHO) supplement has been widely investigated in nondiabetic patients undergoing a variety of surgeries. It has been proved that preoperative CHO could alleviate postoperative insulin resistance (IR) and improve patients’ well-being in nondiabetic patients. However, it remains controversial whether preoperative CHO could yield similar effects in diabetic patients. Till now, seldom has the administration of preoperative CHO been investigated in diabetic patients and there are limited studies reporting IR and postoperative recovery of diabetic patients undergoing cardiac surgery.

**Methods and analysis:**

We present a prospective, single-center, single-blind, randomized, no-treatment controlled trial of preoperative CHO on diabetic patients undergoing off-pump coronary artery bypass grafting (OPCAB). A total of 62 patients will be enrolled and randomized to either Group CHO or Group control (CTRL). Patients in Group CHO will consume CHO fluid containing 50 g carbohydrates orally the evening before surgery (20:00–24:00) while their counterparts in Group CTRL will be fasted after 20:00 the evening before surgery. The primary endpoint is postoperative IR assessed via homeostasis model assessment (HOMA). The secondary endpoints are postoperative levels of potential mediators relating to IR including inflammatory factors and stress reaction characterized by serum cortisol. Exploratory endpoints are in-hospital clinical endpoints. Continuous variables will be compared by Student’s *t*-test or Mann-Whitney *U* test. Categorical variables will be compared with *χ*^2^ test or Fisher’s exact test. All tests in the present study are two-tailed and *P*<0.05 is considered statistically significant. All analyses will be performed with R 4.0.4.

**Discussion:**

This is the first prospective randomized controlled trial of preoperative CHO in diabetic patients undergoing cardiac surgery, with the hypothesis that preoperative CHO could improve postoperative IR and promote postoperative recovery. The research may assist in improving the clinical outcomes of diabetic patients undergoing OPCAB.

**Trial registration:**

The trial has been prospectively registered with ClinicalTrials.gov (https://register.clinicaltrials.gov) and Chinese Clinical Trial Registry (http://www.chictr.org.cn). Registry number is NCT05540249 and ChiCTR2000029664 respectively. Registered on Sept. 14, 2022.

**Clinical trials unit:**

Fuwai Hospital, National Center for Cardiovascular Diseases, Chinese Academy of Medical Sciences & Peking Union Medical College, Beijing, China.

**Supplementary Information:**

The online version contains supplementary material available at 10.1186/s13063-022-07042-w.

## Background

Introduced by a group of European surgeons in 2001, the Enhanced Recovery After Surgery (ERAS) program, as a paradigm shift in perioperative care, has been widely implemented in several surgical subspecialties [1]. Optimizing preoperative fasting protocol is one of the critical elements in the ERAS program. Traditional surgery often requires 8–12 h of preoperative fasting to reduce the risk of pulmonary aspiration of gastric contents. However, it has been proved that fasting leads to a catabolic metabolic state characterized by increased insulin resistance (IR) and post-operative stress reaction [2]. Meanwhile, fasting causes patient discomfort and may be associated with poor clinical outcomes. To ameliorate the deleterious effects caused by the preoperative fasting, the ERAS protocol recommends preoperative oral carbohydrates (CHO) loading for patients undergoing surgery, adults and children alike [3]. Previous study illustrated that the intake of CHO 2 to 3 h before elective colorectal surgery could reduce postoperative IR [4]. Similar effects have also been recognized in other surgeries, such as hepatic surgery [5], hip replacement surgery [6], and cholecystectomy [7].

The prevalence of diabetes mellitus (DM) in Chinese mainland has been increasing these years and is now approximately 12.8% [8]. IR plays a pivotal role in the initiation and development of type 2 diabetes mellitus (T2DM) [9]. Typically, IR is defined as decreased sensitivity or responsiveness to the metabolic effects of insulin [10]. Evidence has suggested that IR could hinder recovery postoperatively and prolong the length of hospital stay [11, 12]. Notably, the degree of postoperative IR is more pronounced after major surgeries than the minor ones [13]. Cardiac surgery represents a typically invasive intervention with a protracted recovery phase that often requires intensive rehabilitation and postoperative pain management. T2DM is a well-established risk factor of coronary artery disease and as much as 20% - 30% of patients undergoing coronary artery bypass grafting are complicated with T2DM [14]. Thus, it is of great significance to investigate the best ERAS protocol for T2DM patients referred to coronary surgery.

The benefits of preoperative CHO in nondiabetic patients have been well-established. In contrast, it remains controversial whether preoperative CHO supplement could yield similar effects in T2DM patients. Till now, the administration of preoperative CHO in T2DM patients has been seldomly investigated and there are limited studies reporting IR and postoperative recovery of diabetic patients undergoing cardiac surgery. Therefore, we put forward the present protocol to introduce a randomized controlled trial examining the impact of preoperative oral CHO on IR and postoperative recovery in T2DM patients undergoing off-pump coronary artery bypass grafting (OPCAB).

## Methods and analysis

### Objectives

The trial is designed to assess the effects of preoperative CHO in T2DM patients undergoing OPCAB. The main hypothesis is that preoperative CHO has advantages in improving postoperative IR and promoting postoperative recovery in T2DM patients undergoing OPCAB. The primary objective is to determine the efficacy of preoperative CHO in improving postoperative IR. The secondary objective is to assess the postoperative level of potential IR-related mediators including biochemical parameters, inflammatory mediators, and stress reaction. In the explorative study, the effects of preoperative CHO on postoperative recovery will be investigated. Postoperative recovery could be assessed via postoperative clinical outcomes such as nausea or vomiting, perioperative myocardial injury or infarction, chest tube drainage, mechanical ventilation time, ICU length, and hospital stay.

### Study setting and trial design

The present study is a single-center, single-blind, randomized controlled trial. The study protocol has been approved by the Ethics Committee of Fuwai Hospital (No. 2019-1301) and has been registered with ClinicalTrials.gov (https://register.clinicaltrials.gov) and Chinese Clinical Trial Registry (http://www.chictr.org.cn) prospectively. Registry number is NCT05540249 and ChiCTR2000029664 respectively.

The relevant data of T2DM patients admitted to the cardiovascular ward will be collected prospectively and consecutively from the electronic medical system. The study will be carried out under strict supervision based on our center’s fasting routine before surgery. Included patients will be randomized into one of the two groups: the control group (Group CTRL) and the preoperative CHO group (Group CHO). The surgeons, anesthesiologists, intensive care unit (ICU) staff, nurses, outcome assessors, data collectors, and data analysts will be blinded to patient grouping. This study is structured according to the 2013 Standard Protocol Item Recommendations for Interventional Trials (SPIRIT) guideline [15] defining standard protocol items for clinical trials and the SPIRIT checklist has been completed (seen in Additional file).

### Eligibility criteria

Trial eligibility criteria will be applied strictly to enroll those T2DM patients undergoing OPCAB. As the cardiopulmonary bypass (CPB) could elicit substantial metabolic changes [16], all included patients will be operated without CPB. As some hormones may affect the IR, we will exclude patients on preoperative hormonal drugs due to endocrine system disease such as thyroid or adrenal dysfunction. All qualified patients will be fully informed of the trial procedures and provide the written informed consent. The detailed eligibility criteria are listed in Table 1.

**Table 1:** Eligibility criteria

Inclusion criteria	
1. Previously diagnosed T2DM 2. Diagnosed with CAD with coronary angiography and indicated for OPCAB 3. Age between 18 and 75 years old 4. First operation in the morning and anesthesia induced around 8:00 5. Written informed consent by the patients	
Exclusion criteria	
1. Combined with other heart diseases or vascular malformations that require surgery in addition to OPCAB 2. Presence of symptoms or signs of heart failure such as orthopnea, distended jugular vein, and lower extremity edema 3. Reduced LVEF (lower than 50%) 4. Combined with gastroesophageal reflux 5. Combined with thyroid insufficiency requiring replacement therapy with levothyroxine 6. Combined with adrenal insufficiency requiring replacement therapy with corticosteroids 7. Refuse to participate.	

*T2DM* type 2 diabetes mellitus, *CAD* coronary artery disease, *OPCAB* off-pump coronary artery bypass graft, *LVEF* left ventricular ejection fraction

### Patient and public involvement statement

While designing the study, we visited an in-hospital DM patient ready to receive the OPCAB. With the assistance of study staff, he reviewed the original study protocol and engaged in discussing about the amendment and optimization of the study protocol.

### Grouping and allocation concealment

Patients meeting the eligibility criteria will be randomized to either the Group CHO or the Group CTRL. The carbohydrates beverage Outfast® (Yichang Humanwell Pharmaceutical Co., Ltd., China) used in the study, is isotonic with osmotic pressure of 280–320mmol/L and PH of 3.8–4.3. Patients in Group CHO will orally consume CHO 355ml containing 50 g of carbohydrates 8–12 h before operation (20:00–24:00 the evening before operation). In order to improve adherence to intervention protocol, patients in the Group CHO will be asked to return the empty beverage bottle to the investigator after drinking the beverage. The Group CTRL will consume no food or drink 12 h before operation (20:00 the evening before operation). Based on the random numbers generated by the computer, qualified patients will be randomized to the Group CHO or Group CTRL the day before surgery. The random number list will be kept in the hand of a dedicated person who will assign participants to Group CTRL or Group CHO according to the random numbers. The surgeons, anesthesiologists, ICU staff, nurses, outcome assessors, data collectors, and data analysts will be blinded to the grouping. Unblinding is permissible when patients suffer from gastric acid reflux and aspiration.

### Anesthesia induction, maintenance, and surgical procedures

Invasive blood pressure will be monitored continuously via the left radial artery once the patients’ arrival at the operating room. Before anesthesia induction, gastric emptying will be assessed with a Philips CX50 ultrasound system equipped with a C5-1 abdominal transducer. The cross-sectional area of the stomach antrum will be measured in the supine position. The image of the stomach antrum will be acquired in the parasagittal plane of the epigastric area.

Anesthesia induction and maintenance will be practiced in a standard manner in all eligible patients. Anesthesia induction consists of a combination of sufentanil 2 μg/kg, midazolam 0.1 mg/kg, etomidate 0.2 mg/kg, and cis-atracurium besylate 0.15 mg/kg. Anesthesia will be maintained with propofol 2 mg/kg/h, dexmedetomidine 0.5 μg/kg/h, cis-atracurium besylate 0.1 mg/kg/h, and sevoflurane 1%. The intraoperative anesthesia management in our center has been previously described in detail [17]. All patients will undergo OPCAB, and the procedures will be performed by the experienced surgeons who have ever performed more than 500 OPCABs. Left internal mammary artery (LIMA) will be routinely grafted to the left anterior descending artery (LAD), and other lesioned coronary arteries will be grafted by single or sequential saphenous vein grafts (SVG). Sometimes radial artery will be harvested if SVG is in poor condition and not suitable for grafting.

### Blood glucose monitoring and management

In this study, the target of glycemic control will be set at 7.8–10 mmol/L.

Blood glucose will be routinely measured every hour during surgery and every 2 h in the ICU after surgery. The blood glucose threshold of initiating insulin therapy will be set at 10.0 mmol/L for the critically ill patients in the ICU. If the blood glucose is beyond 10 mmol/L but below 12.2mmol/L, a bolus dose of 2–4U insulin will be administered intravenously and the initial maintaining rate will be set at 2U/h. If the blood glucose is beyond 12.2mmol/L but below 16.7mmol/L, a bolus dose of 4–6U insulin will be administered intravenously and the initial maintaining rate is set at 4U/h. If the blood glucose is beyond 16.7mmol/L, a bolus dose of 6–8U insulin will be administered intravenously and the initial maintenance rate is set at 6U/h. If the blood glucose continues to rise, the infusion rate will be raised with the increment of 2U/h. If the blood glucose decreases by more than 25%, the rate of intravenous insulin infusion will be reduced with the decrement of 2U/L. If the blood glucose is less than 5.6 mmol/L, 20–40mL 50% glucose solution will be intravenously infused to restore the blood glucose to 7.8–10 mmol/L.

After patients return to the surgical ward, fingertip blood glucose will be routinely measured four times a day, that is, fasting blood glucose in the morning and 2 h after three meals. As patients have resumed regular diet, glycemic management will be turned to preoperative medical protocol, whether oral hypoglycemic drugs, insulin, or both. If the blood glucose rises sharply beyond 10.0mmol/L, extra insulin will be administered percutaneously to restore the blood glucose to the target level.

### Data collection, management, and monitoring

A well-designed questionnaire will be applied to collect the baseline characteristics of eligible patients. Two to 3 persons will be trained to assist in collecting baseline characteristics including demographical data and medical history. The results of blood tests will be obtained from the digital medical record system. A dedicated person will record all the data in an excel sheet and no one else will have access to the data in the excel sheet until statisticians analyze the collected data at the end of the study. As the intervention in the present study is low-risk and unlikely to cause severe complications to patients, the Independent Data Monitoring Committee is not considered.

### Quality control

Before the enrollment of the first patient, all participants in the trial including surgeons, anesthesiologists, ICU staff, nurses, outcome assessors, data collectors, and data analysts shall receive unified training to be familiar with the detailed process of the trial. This trial steering committee (TSC) is composed of the chief supervisor and five members with more than 5-year experience in clinical trial. With the assistance of the five members, the chief supervisor shall organize meetings to discuss problems in the process of the trial every 2 weeks and oversee the conduct and progress of the trial. The five members shall coordinate the work of involved departments and provide the necessary support for the trial.

### Statistical analysis

Modified intention-to-treat analysis is adopted to analyze the data. If the operation of an enrolled patient is canceled due to special circumstances such as fever, the random number will be reserved to him/her until the end of the trial. If the patient whose operation is canceled contemporarily is operated later before the end of the trial, he/she will be assigned to the Group CTRL or Group CHO according to the early-assigned random number. If the patient who doesn’t undergo the operation as planned is not operated before the end of the trial or whose operation is rescheduled to a later time of the day instead of the first operation at 8’oclock, he/she will be excluded from the trial and extra random number will be generated to enroll enough patients to the prespecified sample size.

The primary endpoints, homeostasis model assessment (HOMA) of IR, will be compared between the two groups at several fixed time after surgery. In addition, overall postoperative IR will be assessed via area-under-curve of HOMA (AUC_HOMA_) and AUC_HOMA_ will be compared between the two groups. The secondary endpoints, postoperative IR-related mediators, will be compared between the two groups primarily at the first morning after surgery. In the exploratory study, in-hospital outcomes as listed in the following part of the study endpoints will be compared between the two groups. Normal distribution of continuous variables will be tested using the Shapiro-Wilks test. Continuous variables will be presented as mean ± standard deviation if the variables follow a normal distribution, otherwise median and interquartile range. Categorical variables will be presented as number and percentage. Missing data will be managed via multiple imputation. Continuous variables with normal distribution will be compared by Student’s *t*-test between the groups. Continuous variables with skewed distribution will be compared by Mann-Whitney *U* test. We will compare categorical variables with the *χ*^2^ test or Fisher’s exact test. The Spearman rank correlation test will be utilized to assess the correlation between continuous variables.

The analysis will be performed by a dedicated data analyst who is masked to subjects’ group allocation. All the tests in the present study are two-tailed and *P*<0.05 is considered statistically significant. All analyses will be performed with R 4.0.4 (R Foundation for Statistical Computing, Vienna, Austria).

### Sample size calculation

The primary outcome is the postoperative IR assessed *via* HOMA of the function of islet β cell (HOMA-β). Previous literature [18] showed that the HOMA-β of the CHO group tended to be higher than that of the F group-β (87% *vs*. 47.5%). In the present study, the test power was set to 0.80, two-sided α was set to 0.05, and the dropout rate was set to 0.1. It was estimated that a total of 62 patients with 31 patients in group CHO and 31 patients in group C would be enrolled.

## Study endpoints

### Primary endpoints

The primary endpoints are postoperative HOMA-β and HOMA-IR. HOMA score is a mathematical model that can reflect the balance between liver glucose output and insulin secretion, and it has been previously demonstrated that it has a strong correlation with clamp-measured total glucose disposal, the gold standard of assessing IR [19].

The calculation formulas of HOMA-β and HOMA-IR are as follows:

$$\textrm{HOMA}-\upbeta \%=\left[20\times \textrm{fasting}\ \textrm{insulin}\ \left(\textrm{mu}/\textrm{L}\right)\right]/\left[\textrm{fasting}\ \textrm{blood}\ \textrm{glucose}\ \left(\textrm{mmol}/\textrm{L}\right)-3.5\right]\%$$

$$\textrm{HOMA}-\textrm{IR}=\left[\textrm{blood}\ \textrm{insulin}\ \left(\textrm{mu}/\textrm{L}\right)\times \textrm{Blood}\ \textrm{glucose}\ \left(\textrm{mmol}/\textrm{L}\right)\right]/22.5$$

The HOMA-β is expressed as percentage, and its normal value is 100%. The HOMA-IR is expressed without unit, and its normal value is 1. With the increase of IR, the HOMA-β will be lower than normal and the HOMA-IR will be higher than normal.

**Fig. 1:**
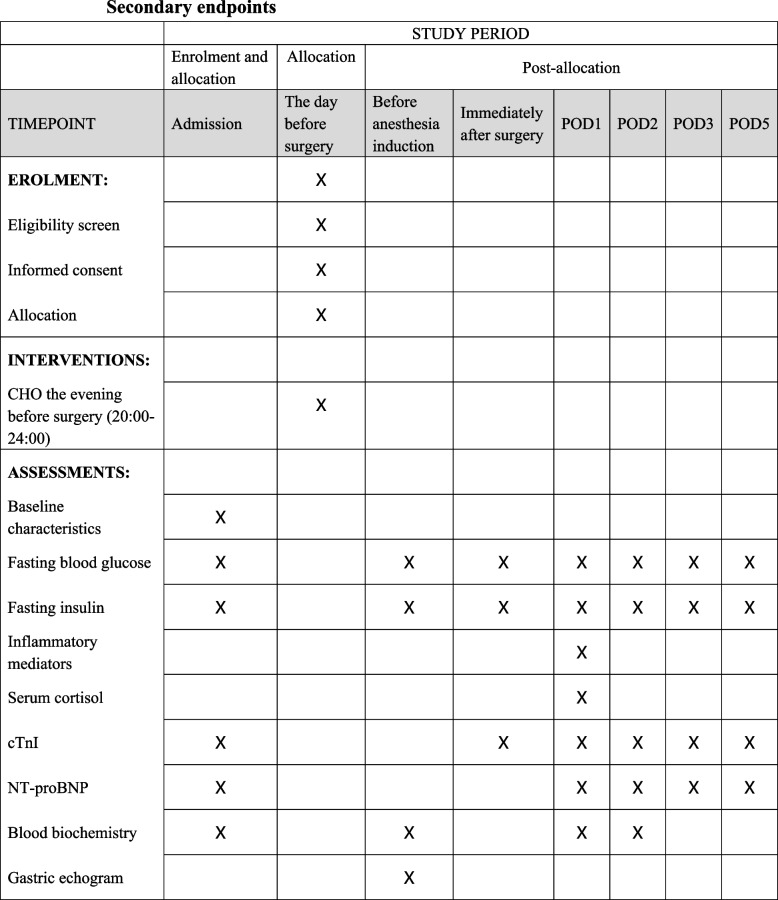
SPRIRIT figure

The baseline HOMA-β and HOMA-IR will be acquired at admission. And then these two HOMA scores will be sequentially measured before anesthesia induction, immediately after surgery, the first morning after surgery (POD1), POD2, POD 3, and POD 5 (Fig. 1)

### Secondary endpoints

The secondary endpoints are postoperative levels of potential mediators relating to IR. First, biochemical parameters include fasting blood glucose and fasting blood insulin. Second, inflammatory mediators and stress reaction will be assessed the first morning after surgery. Inflammatory mediators include interleukin-1 (IL-1), interleukin-6 (IL-6), interleukin-8 (IL-8), interleukin-10 (IL-10), tumor necrosis factor-α (TNF-α), leukocyte, and high-sensitivity C-reactive protein (hs-CRP). The degree of stress reaction could be reflected to some extent by the serum cortisol level (Fig. 1).

### Exploratory endpoints

Exploratory outcomes are in-hospital clinical outcomes after surgery. In-hospital clinical outcomes after surgery include nausea or vomiting, perioperative myocardial injury or infarction, intra-aortic balloon pump (IABP) or extracorporeal membrane oxygenation (ECMO), new-onset atrial fibrillation (POAF), stroke, delirium, acute kidney injury (AKI), infection, blood transfusion, chest tube drainage, reoperation for bleeding, mechanical ventilation time, ICU length, and length of hospital stay.

The severity of perioperative myocardial injury is assessed via peak high-sensitivity cardiac troponin I (cTnI). The diagnosis of perioperative myocardial infarction is determined using a combination of biomarkers, echocardiographic signs of wall-motion abnormality, and new Q wave on ECG [20]. POAF is defined as new-onset atrial fibrillation lasting at least 10min on the electrocardiogram (ECG) monitor or atrial fibrillation that requires treatment with medication after surgery. Perioperative stroke is identified via neurological symptoms, signs, and imaging findings of new high-density or low-density shadows on computed tomography (CT). Delirium is identified via the Confusion Assessment Method for the Intensive Care Unit (CAM-ICU) [21]. AKI is defined as an increase in serum creatine by more than 26.5μmol/L within 48 h or an increase in serum creatine to higher than 1.5 times baseline within the postoperative 7 days according to the Kidney Disease: Improving Global Outcomes (KDIGO) clinical guidelines [22]. Surgical site infection in this study includes superficial sternal wound infection, deep sternal wound infection, and harvest site infection. Pulmonary infection is diagnosed based on typical clinical symptoms and signs, chest X-ray, positive culture of sputum or tracheobronchial secretions, and elevated white blood cell counts. Blood transfusion is strictly supervised and follows the indication of blood transfusion published by our center [23]. Reoperation is indicated if there is more than 200mL drainage for more than 3 h or active bleeding is considered from surgical perspective.

## Discussion

This study is a randomized, single-center, parallel-group trial aiming to investigate the effect of preoperative CHO on the postoperative IR and in-hospital clinical outcomes. As previously mentioned, preoperative CHO has been proven to improve IR after surgery in nondiabetic patients but the relevant evidence on T2DM patients is scarce. Thus, we will perform the research, especially in T2DM patients undergoing OPCAB.

As the degree of postoperative IR is related to the severity of surgical trauma [24], cardiovascular surgery can be a good model to investigate the effect of preoperative CHO on postoperative stress response as well as IR after major surgery with substantial trauma. Considering the substantial effect of CPB on metabolic state and IR, we select patients undergoing OPCAB to avoid the effect of CPB on the results. Standard preoperative CHO protocol in the nondiabetic population comprises of 800mL CHO the evening before surgery and 400mL 2–3 h before surgery [25]. However, we omit the CHO supplement 2–3 h before surgery. First, this trial is the first attempt to supply T2DM patients with CHO before OPCAB in our center and we establish the trial as a step stone for bringing our center up to date with the ERAS recommendations. Thus, we omit the seemingly aggressive intervention of CHO supplement 2–3 h before surgery in these high-risk T2DM patients at this step. In the future, we’ll make a further attempt of adding CHO supplement 2–3 h before surgery based on the results of this trial. Second, patients in the study have severe coronary artery disease and some of them may have unstable angina pectoris. Consumption of too much liquid might increase the preload and induce angina pectoris. Third, intraoperative hyperglycemia could increase the risk of complications after cardiac surgery [26]. CHO supplement 2–3 h may induce intraoperative hyperglycemia in T2DM patients with inherent glycemic disorders, thereby incurring additional risks to patients. Thus, from the perspective of safety, the intervention in the Group CHO is designed to supplement CHO only once the evening before surgery. The reason why we select patients who undergo the first operation every day is to ensure a fixed preoperative fasting time and increase comparability between the two groups.

The exact mechanism of IR after stress has not been fully understood, and there currently exist two potential explanations: on the one hand, the increased release of catecholamine, growth hormone, glucocorticoid, and tumor necrosis factor in response to surgical trauma causes an increase in liver glycogen release and IR [27]; on the other hand, in peripheral tissues, glucocorticoids and epinephrine reduce glucose uptake, while cytokines such as tumor necrosis factor and interleukin-1 inhibit insulin signal transmission. The lack of insulin signal receptor and glucose transporter 4 leads to decreased glucose uptake and IR [28, 29]. In the present study, we will compare the degree of stress response by measuring the serum cortisol level and postoperative inflammation response by serum inflammatory factors.

In summary, this is the first prospective randomized controlled trial of preoperative CHO in T2DM patients undergoing OPCAB, with the hypothesis that preoperative CHO could improve postoperative IR and promote postoperative recovery. In the future, the feasibility, safety, and efficacy of CHO supplementation 2–3 h before surgery in T2DM patients warrant further investigation.

## Trial status

The trial hasn’t started patient enrollment at the time of manuscript submission. The trial plans to enroll the first patient in November 2022 and end in January 2023.

## Supplementary Information

**Additional file 1.**

## Data Availability

The final trial data for this protocol could be supplied on reasonable request.

## Abbreviations

AKI: Acute kidney injury
CAD: Coronary artery disease
CAM-ICU: Confusion Assessment Method for the Intensive Care Unit
CHO: Oral carbohydrates
CPB: Cardiopulmonary bypass
CT: Computed tomography
cTnI: Cardiac troponin I
DM: Diabetes mellitus
ECG: Electrocardiogram
ECMO: Extracorporeal membrane oxygenation
ERAS: Enhanced recovery after surgery
HOMA: Homeostasis model assessment
hs-CRP: High-sensitivity C-reactive protein
IABP: Intra-aortic balloon pump
ICU: Intensive care unit
IR: Insulin resistance
IL: Interleukin
KDIGO: Kidney Disease: Improving Global Outcomes
LAD: Left anterior descending artery
LIMA: Left internal mammary artery
LVEF: Left ventricular ejection fraction
NDM: Non-diabetes mellitus
OPCAB: Off-pump coronary artery bypass grafting
POAF: New-onset atrial fibrillation
SPIRIT: Standard Protocol Item Recommendations for Interventional Trials
SVG: Saphenous vein graft
T2DM: Type 2 diabetes mellitus
TNF-α: Tumor necrosis factor-α
